# Uterine artery Doppler flow velocimetry parameters for predicting gestational trophoblastic neoplasia after complete hydatidiform mole, a prospective cohort study

**DOI:** 10.6061/clinics/2017(05)05

**Published:** 2017-05

**Authors:** Flavia Tarabini Castellani Asmar, Antonio Rodrigues Braga-Neto, Jorge de Rezende-Filho, Juliana Marques Simões Villas-Boas, Rafael Cortés Charry, Izildinha Maesta

**Affiliations:** I Departamento de Ginecologia e Obstetricia, Faculdade de Medicina de Botucatu, UNESP – Universidade Estadual Paulista, Botucatu, SP, BR; II Centro de Doenças Trofoblásticas, Maternidade-Escola da Universidade Federal do Rio de Janeiro, Rio de Janeiro, RJ, BR; III Centro de Doenças Trofoblásticas, Hospital Universitário Antônio Pedro, Universidade Federal Fluminense, Niterói, RJ, BR; IV Departamento de Ginecologia e Obstetricia, Faculdade de Medicina, Universidade Federal do Rio de Janeiro, Rio de Janeiro, RJ, BR; V Centro de Doenças Trofoblásticas, Faculdade de Medicina de Botucatu, UNESP – Universidade Estadual Paulista, Botucatu, SP, BR; VI Department of Gynecology and Obstetrics, GTD unit, University Hospital of Caracas, Central University of Venezuela, Caracas, Venezuela

**Keywords:** Complete Hydatidiform Mole, Uterine Artery Doppler Flow Velocimetry, Gestational Trophoblastic Neoplasia

## Abstract

**OBJECTIVES:**

Doppler ultrasonography can be used to assess neoangiogenesis, a characteristic feature of postmolar gestational trophoblastic neoplasia. However, there is limited information on whether uterine artery Doppler flow velocimetry parameters can predict gestational trophoblastic neoplasia following a complete hydatidiform mole. The purpose of this study was as follows: 1) to compare uterine blood flow before and after complete mole evacuation between women who developed postmolar gestational trophoblastic neoplasia and those who achieved spontaneous remission, 2) to assess the usefulness of uterine Doppler parameters as predictors of postmolar gestational trophoblastic neoplasia and to determine the best parameters and cutoff values for predicting postmolar gestational trophoblastic neoplasia.

**METHODS:**

This prospective cohort study included 246 patients with a complete mole who were treated at three different trophoblastic diseases centers between 2013 and 2014. The pulsatility index, resistivity index, and systolic/diastolic ratio were measured by Doppler flow velocimetry before and 4-6 weeks after molar evacuation. Statistical analysis was performed using Wilcoxon’s test, logistic regression, and ROC analysis.

**RESULTS:**

No differences in pre- and post-evacuation Doppler measurements were observed in patients who developed postmolar gestational trophoblastic neoplasia. In those with spontaneous remission, the pulsatility index and systolic/diastolic ratio were increased after evacuation. The pre- and post-evacuation pulsatility indices were significantly lower in patients with gestational trophoblastic neoplasia (odds ratio of 13.9-30.5). A pre-evacuation pulsatility index ≤1.38 (77% sensitivity and 82% specificity) and post-evacuation pulsatility index ≤1.77 (79% sensitivity and 86% specificity) were significantly predictive of gestational trophoblastic neoplasia.

**CONCLUSIONS:**

Uterine Doppler flow velocimetry measurements, particularly pre- and post-molar evacuation pulsatility indices, can be useful for predicting postmolar gestational trophoblastic neoplasia.

## INTRODUCTION

A complete hydatidiform mole (CHM), which is characterized by increased hyperplasia, progresses to gestational trophoblastic neoplasia (GTN) in 9-20% of cases 1. Suction curettage is the method of choice for CHM evacuation. During CHM follow-up, serial assessment of serum human chorionic gonadotropin (hCG) is the standard method to identify postmolar malignancy. A rise or plateau in hCG indicates the likely onset of malignancy requiring chemotherapy.

Predicting CHM malignancy potential is of the utmost importance because it allows for the prompt treatment of post-molar GTN, limiting the exposure of most patients to combination chemotherapy 2. Moreover, predicting CHM malignant potential can help in selecting the patients who may benefit from prophylactic chemotherapy, particularly when close hCG follow-up is not possible.

Clinical factors, such as age, ovarian enlargement, a large-for-date uterus, previous history of molar pregnancy, hCG level 3,4, and expression of endocrine gland-derived vascular endothelial growth factor (EG-VEGF), HIF-1alpha, and TGF- beta 3 have all been reported to be helpful in predicting post-CHM GTN 5,6.

Transvaginal ultrasound with power Doppler can predict delayed response to chemotherapy and drug resistance in low-risk trophoblastic neoplasia 7. Doppler US of the pelvis, an important tool in the diagnosis of GTN 8,9, is also used as part of GTN routine staging to assess the uterine volume and blood flow. Changes in flow resistance can reliably determine the appropriate chemotherapeutic regimen for GTN as well as predict the GTN response to chemotherapy 10,11. Moreover, the highly abnormal flow patterns seen in invasive gestational trophoblastic neoplasia can be measured by Doppler US as early as two weeks post-evacuation, which is before the appearance of lesions 12 or an hCG plateau or rise 10. However, it remains controversial whether uterine artery Doppler flow velocimetry (DFV) parameters can predict GTN following CHM 13.

The purpose of this study was two-fold and is summarized as follows: 1) to compare uterine blood flow before and after CHM evacuation between women with post-molar GTN and those with spontaneous remission 2) to assess the usefulness of uterine DFV parameters as predictors of post-CHM GTN and determine the best prediction parameters and cutoffs.

## METHODS

This prospective cohort study included women with CHM who were treated at three different trophoblastic diseases centers between 2013 and 2014. CHM was diagnosed based on ultrasound findings confirmed by histopathological analysis and p57 immunohistochemistry. All patients were initially treated with suction D&C, and they underwent uterine artery Doppler flow velocimetry (DFV) at admission and after CHM evacuation. Women were excluded from the study if they did not undergo post-evacuation DFV or were lost to follow-up after this procedure. The study was approved by the Institutional Committee of Research Ethics (August 8, 2013, #15983613.8.0000.5411), and it was conducted in accordance with the Declaration of Helsinki. Written informed consent was obtained from all patients.

Assuming that GTN follows CHM in 15% of cases, the minimum sample size was estimated to be 175 patients (type 1 error = 0.05 and type 2 error = 0.20) to achieve a statistical power of 0.8 (80%) in all tests.

Data collected at admission included age, number of pregnancies, parity, gestational age at diagnosis, uterine size, presence of theca lutein cysts >6 cm (indicative of higher risk of malignancy), and pre-evacuation hCG level. An excessive uterine size was considered present if the uterus was at least 4 weeks greater in size than expected for the gestational age on clinical examination 14,15. Theca lutein ovarian cysts >6 cm were identified by pelvic ultrasound and by bimanual pelvic examination performed under anesthesia prior to uterine evacuation 14.

Transabdominal sonography was performed for global evaluation of the pelvis, and transvaginal imaging was performed for a more detailed evaluation of the uterus, endometrial cavity and adnexae. Because the site of molar implantation in the uterus was unknown, the DFV of both uterine arteries was performed at admission (up to 2 hours pre-evacuation) and 4-6 weeks after CHM evacuation. Doppler assessments were performed using a Colored Doppler Ultrasound (Power Vision 6000 TOSHIBA^®^, New York, NY, USA) with a 5.0-MHz convex transducer. For the transvaginal scan, women were asked to empty their bladders and were placed in the dorsal lithotomy position. The ultrasound probe was then inserted into the vagina and placed in the lateral fornix, and the uterine artery was identified using color Doppler at the level of the internal cervical os. The ascending branch of the uterine artery at both its paracervical portion and at the point closest to the internal os was identified, and pulsed wave Doppler was applied with the sampling gate set at 2 mm at an angle of insonation <30°. After four consecutive similar waveforms were obtained, the pulsatility index (PI), resistivity index (RI), and systolic/diastolic ratio (S/D) were automatically calculated by the scanner software. The PI, RI and S/D were defined by the following formulas:

PI= A-B/Mean

RI= A-B/A

SD= A/B

where:

A= peak systolic velocity

B= end diastolic velocity

Mean= mean velocity

Post-molar GTN was diagnosed according to the following criteria standardized by the International Federation of Gynecology and Obstetrics (FIGO 2002) 16:

1. An hCG level plateau of four values ±10% recorded over a 3-week duration (days 1, 7, 14, and 21).
2. An hCG level increase of more than 10% of three values recorded over a 2-week duration (days 1, 7, and 14).

Remission was declared after undetectable hCG levels were achieved for 3 consecutive weeks and then at monthly intervals for 1 year.

Statistical analyses were performed using SPSS v 21.0(IBM Corp, Armonk, NY, USA). The non-parametric Wilcoxon test was used to compare pre- and post- CHM evacuation DFV parameters between women who developed post-molar GTN and those who achieved spontaneous remission. The usefulness of uterine DFV parameters as predictors of post-CHM GTN was assessed using multiple logistic regression adjusted for age, gestational age, enlarged uterus for gestational age, theca lutein cysts, and pre-evacuation hCG level. The best DFV parameters and cutoff values for predicting post-CHM GTN were determined by receiver operating characteristic (ROC) analysis, including calculation of the areas under the curves. Statistical significance was set at *p*<0.05.

## RESULTS

Of 276 women diagnosed with CHM, 246 were included in the analysis. Of the 30 remaining women, 17 did not undergo post-evacuation DFV, and 13 were lost to follow-up after post-evacuation DFV; therefore, they were excluded from the study. The median patient age was 24 years (13-47 years), and the median gestational age was 8 weeks (4-16 weeks). No significant differences were observed between pre- and post-evacuation or right and left uterine artery DFV measurements. Development into GTN occurred in 20% of the cases (Table 1).

Table 2 shows that, compared to the pre-evacuation measurements, the PI and S/D significantly increased in both arteries, while the RI remained unchanged after evacuation in women with spontaneous remission. In those who developed GTN, the DFV measurements remained unchanged pre- and post-evacuation.

PI was the Doppler parameter that was most strongly associated with risk of post-CHM GTN both pre-and post-evacuation, i.e., women showing lower PI values were more likely to develop GTN following CHM (OR 13.9-30.5, 95% CI = 5.97 – 32.60 and 12.01 – 77.52, respectively) (Table 3). The best PI cutoff points for predicting GTN were ≤1.38 pre-evacuation (77% sensitivity and 82% specificity; 95% CI 0.72 – 0.86) and ≤1.77 post-evacuation (79% sensitivity and 86% specificity; 95% CI 0.77 – 0.91) (Table 4).

## DISCUSSION

Neoangiogenesis is a critical feature of malignancy. It is a key component of tumorigenesis, tumor growth and metastatic spread, and it is associated with drug resistance and poor prognosis in several solid tumors 11,17.

Doppler flow velocimetry (DFV) offers a noninvasive technique for neoangiogenesis quantification by assessing the vascularity and circulatory resistance of blood flow through the uterine arteries. In the early diagnosis of invasive GTD, DFV clearly shows an advantage over classical hCG measurements because it can detect the presence of post-evacuation invasive GTD weeks before an hCG rise or plateau occurs 10,18. Doppler uterine artery PI is inversely proportional to tumor vascularity, and a low uterine artery PI indicates increased arteriovenous shunting 11, a feature that leads to large low-resistance blood vessels, an inherent characteristic of trophoblastic invasion.

Our results show that most women participating in this study were young (13-47 years) and were diagnosed in the early first trimester of pregnancy. The development of post-CHM GTN occurred in 20% of the cases; this rate is similar to those found in studies that include patients diagnosed later in the first trimester or even in the second trimester of pregnancy 19-21. This indicates that the gestational age at diagnosis does not affect the risk of developing postmolar GTN, as demonstrated by others 15,22,23.

Compared to pre-evacuation measurements, the PI and S/D significantly increased (in both arteries) after evacuation in participants with spontaneous remission (n=197). In those who developed GTN, the pre- and post-evacuation DFV measurements remained (n=49) significantly lower. Similar findings 15,18,24,25 have been reported in smaller patient populations. Yalcin et al. 18 found that significantly lower uterine artery Doppler indices before molar evacuation were associated with the development of GTN in 5 compared with 16 patients with spontaneous remission. Similarly, Gungor et al. 26 reported a lower uterine artery RI before molar evacuation in 12 patients who developed persistent malignant disease compared with 20 patients who had spontaneous remission. In contrast, the study by Chan et al. 27 showed no difference in RI in 11 patients with spontaneous remission compared with 21 patients with postmolar persistent disease.

In this study, PI was the Doppler parameter that was most strongly associated with the risk of post-CHM GTN pre-and post-evacuation. The best cutoff PI values for predicting GTN were ≤1.3 pre-evacuation (77% sensitivity, 82% specificity) and ≤1.7 post-evacuation (79% sensitivity, 86% specificity). These values are similar to those reported by Maymon et al. 12, who observed that PI levels ≤1.5 predicted GTN in the 2nd post-evacuation week. Notably, a cutoff ≤1.1 has been associated with chemoresistance in trophoblastic tumors 11,. The high sensitivity and specificity values obtained in this study support the theory that DFV measurements can identify those patients at a higher risk of developing GTN after CHM.

Previous studies assessing the role of Doppler indices in predicting the development of trophoblastic tumors have yielded conflicting findings, which is probably because of the small number of patients. The results of this larger prospective study show that uterine DFV measurements, particularly pre- and post-molar evacuation PI, can be useful for predicting GTN following CHM in a much larger population. Indeed, participants with lower PI values were more likely to develop GTN following CHM. However, although the Doppler angle of insonation was kept as low as possible, small deviations and inaccuracies in drawing the contour of the waves may have occurred due to the small diameter and tortuosity seen in the uterine vessels. Nonetheless, the uterine artery PI has been considered an objective measure of vascularity that is not based on the subjective selection of regions of interest, and it has been reported as a useful noninvasive marker of tumor vascularity in previous studies 11,29. Interestingly, in other gynecologic conditions, such as endometrial lesions and adnexal masses 31,32, the uterine artery PI has also been used for differentiating between benign and malignant tumors.

Predicting a high chance for developing GTN has become highly important as the early detection of molar pregnancy with ultrasound no longer allows for observation of some clinical features, such as theca lutein cysts and large-for-age uterine size, which are only encountered at a more advanced gestational age 25. Furthermore, hCG testing cannot detect the patients who are more likely to develop GTN in the first weeks after evacuation 25.

It is noteworthy, however, that, according to Kohorn 33, the use of ultrasound or DFV to follow hydatidiform mole regression is clinically and fiscally counterproductive. Nevertheless, several studies have demonstrated that changes in Doppler indices inversely correlate with hCG titers and that ultrasound can accurately identify patients who are at a high risk for post-evacuation GTN when hCG levels are not helpful 15,18,24,25,34. Because serial hCG assessment cannot detect GTN in the first three weeks 25, it can delay diagnosis, which has been associated with an increased FIGO risk score 25 and greater chance of resistant disease 35. The cost-effectiveness of ultrasound technologies has become widely recognized. Moreover, as each one-point increment in the FIGO score is associated with an average increase of 17 days in hCG remission time 36, the early detection of GTN may decrease the economic and social burden of the disease.

In brief, this study demonstrated the following: 1) post-evacuation DFV parameters were significantly increased (in both arteries) in women with spontaneous remission, 2) lower PI values were associated with a higher risk of developing GTN following CHM, and 3) PI was the Doppler parameter most strongly associated with risk of post-CHM GTN with cutoffs of ≤1.3 pre-evacuation and ≤1.7 post-evacuation. These results, which were prospectively obtained in a large population, validate the usefulness of the uterine artery PI as a marker of GTN development following CHM.

## AUTHOR CONTRIBUTIONS

Asmar FT conducted the research and wrote the manuscript. Braga-Neto AR helped selecting patients, performed data collection and critically revised the manuscript. Rezende-Filho J critically revised the manuscript. Villas-Boas JM designed the analytic strategy. Charry RC critically revised the manuscript. Maesta I conceived, designed and supervised the project.

## Figures and Tables

**Table 1:** Patient characteristics (*n* = 246) and pre- and post-evacuation uterine artery DFV measurements.

Variable	
Age (years) (1)	24 (13 – 47)
Gravidity (1)	2 (1 – 16)
Parity (1)	0 (0 – 8)
Gestational age (weeks) (1)	8 (4 – 16)
Uterine Size > gestational age	58 (24%)
Theca lutein cysts	26 (11%)
Pre-evacuation hCG (mUI/mL) (1)	53171 (382 – 2842101)
Right, Pre-evacuation, PI (1)	1.83 (0.73 – 3.34)
Right, Pre-evacuation, RI (1)	0.82 (0.43 – 3.40)
Right, Pre-evacuation, S/D (1)	4.80 (1.88 – 16.40)
Left, Pre-evacuation, PI (1)	1.78 (0.75 – 3.09)
Left, Pre-evacuation, RI (1)	0.82 (0.43 – 1.82)
Left, Pre-evacuation, S/D (1)	4.40 (1.70 – 17.00)
Right, Post-evacuation, PI (1)	2.02 (0.55 – 6.05)
Right, Post-evacuation, RI (1)	0.82 (0.39 – 2.49)
Right, Post-evacuation, S/D (1)	5.90 (1.20 – 19.20)
Left, Post-evacuation, PI (1)	1.94 (0.56 – 4.06)
Left, Post-evacuation, RI (1)	0.83 (0.36 – 0.99)
Left, Post-evacuation, S/D (1)	5.53 (1.68 – 37.10)
Postmolar GTN	49 (20%)

(1) Median (minimum-maximum)

**Table 2:** Pre- and post-evacuation uterine artery Doppler flow velocimetry in women who achieved spontaneous remission and those who developed GTN.

	Spontaneous remission (n=197)			GTN (n=49)		
	Pre	Post	*p* (1)	Pre	Post	*p* (1)
Right, PI	1.87 (0.73 - 3.34)	2.12 (0.55 - 5.24)	<0.001	1.13 (0.77 - 2.36)	1.10 (0.62 - 6.05)	0.778
Right, RI	0.82 (0.47 - 3.40)	0.83 (0.39 - 2.49)	0.648	0.79 (0.43 - 0.92)	0.78 (0.43 - 1.12)	0.536
Right, S/D	4.76 (1.88 - 16.40)	5.9 (1.88 - 19.20)	<0.001	4.9 (2.12 - 12.38)	4.89 (1.20 - 12.7)	0.045
Left, PI	1.89 (0.75 - 3.09)	1.97 (0.68 - 4.06)	<0.001	1.16 (0.80 - 2.95)	1.20 (0.56 - 3.29)	0.375
Left, RI	0.82 (0.43 - 1.82)	0.83 (0.41 - 0.99)	0.236	0.81 (0.52 - 0.98)	0.78 (0.36 - 0.98)	0.186
Left, S/D	4.28 (1.75 - 17.00)	5.5 (1.68 - 13.08)	<0.001	5.8 (1.70 - 12.5)	5.80 (1.81 - 37.1)	0.984

(1) Wilcoxon. Median (minimum-maximum)

**Table 3:** Multiple logistic regression of the usefulness of each uterine DFV parameter as a predictor of gestational trophoblastic neoplasia following development of a complete hydatidiform mole, adjusted for age, gestational age, size uterus > date, theca lutein cysts, and pre-evacuation hCG level.

Side: Time: Index	*p*	OR (95% CI)
Right, Pre-evacuation, PI	0.0000	19.87 (7.60 – 51.91)
Right, Pre-evacuation, RI	0.0480	11.48 (1.02 – 129.26)
Right, Pre-evacuation, S/D	0.0060	1.27 (1.07 – 1.51)
Left, Pre-evacuation, PI	0.0000	13.96 (5.97 – 32.60)
Left, Pre-evacuation, RI	0.3380	2.87 (0.33 – 24.83)
Left, Pre-evacuation, S/D	0.9510	1.00 (0.88 – 1.14)
Right, Post-evacuation, PI	0.0000	16.25 (7.25 – 36.44)
Right, Post-evacuation, RI	0.0430	13.29 (1.08 – 163.54)
Right, Post-evacuation, S/D	0.0760	1.13 (0.98 – 1.29)
Left, Post-evacuation, PI	0.0000	30.51 (12.01 – 77.52)
Left, Post-evacuation, RI	0.0170	24.23 (1.75 – 334.80)
Left, Post-evacuation, S/D	0.1670	0.95 (0.88 – 1.02)

**Table 4:** PI, RI and S/D cutoff values; sensitivity; and specificity for predicting gestational trophoblastic neoplasia following a complete hydatidiform mole.

Side: Time: Index	A (95% IC)	*p*	Cutoff	Se	S
Right, Pre-evacuation, PI	0.79 (0.72 – 0.86)	<0.001	1.38	0.77	0.82
Right, Pre-evacuation, RI	0.67 (0.59 – 0.75)	<0.001	0.79	0.55	0.66
Right, Pre-evacuation, S/D	0.53 (0.43 – 0.62)	0.517	4.89	0.42	0.43
Left, Pre-evacuation, PI	0.76 (0.67 – 0.84)	<0.001	1.35	0.73	0.84
Left, Pre-evacuation, RI	0.54 (0.44 – 0.65)	0.326	0.8	0.47	0.55
Left, Pre-evacuation, S/D	0.40 (0.29 – 0.50)	0.03	4.75	0.36	0.39
Right, Post-evacuation, PI	0.82 (0.74 – 0.90)	<0.001	1.73	0.77	0.84
Right, Post-evacuation, RI	0.61 (0.51 – 0.70)	0.017	0.79	0.57	0.67
Right, Post-evacuation, S/D	0.55 (0.45 – 0.66)	0.209	5.42	0.51	0.58
Left, Post-evacuation, PI	0.84 (0.77 – 0.91)	<0.001	1.77	0.79	0.86
Left, Post-evacuation, RI	0.66 (0.56 – 0.75)	<0.001	0.8	0.59	0.68
Left, Post-evacuation, S/D	0.48 (0.38 – 0.58)	0.706	5.36	0.42	0.53

A=Area under the ROC curve; Se = Sensitivity; and S = Specificity.
